# Parliament and the Profession

**Published:** 1915-10-23

**Authors:** 


### PARLIAMENT AND THE PROFESSION.

### Medical Help from Australia.

Mr. Cathcart Wilson has given notice to ask the Under Secretary of State for War if the Tenth Australian General Hospital arrived here fully equipped with twenty-one medical officers and seventy-one sisters, and if so far it has been found impossible to utilise its services. Assuming this is a correct statement of the facts, Mr. Wilson desires to know who is responsible for such a state of affairs, and if, out of respect for Australian sentiment, the War Office will see that this specially equipped unit will not be split up but employed as a unit.

### Experiments on Living Animals.

In the House of Commons on October 12 Mr. George Greenwood complained of the delay in issuing the return of experiments on living animals for the year 1914, which, by the way, has now been published. Mr. Greenwood's suggestion was that the delay precluded all possibility of instituting any prosecution for any offences that may have been committed during the year. His view was that the return was practically the only means whereby the public could obtain any information regarding irregularities, but the Under Secretary of State for the Home Department reassured Mr. Greenwood on this point by stating that the date of publication of the return does not affect the power of the Home Office to institute proceedings, since any contravention is reported as soon as it is discovered. As a matter of fact the report shows that the few irregularities that have come under notice have been of a trivial technical character.

### The Customs Duty on Chloroform.

As was anticipated, the Finance Bill embodies no provision with respect to the proposed repayment to hospitals of the amount of duty on spirits paid by them. It contains, however, a clause providing that the Customs duties on certain medicinal preparations in the manufacture of which spirit is used shall cease, the articles in question including chloroform, collodion, and chloral hydrate. What effect this concession is likely to have in time of war, except in one or two cases, it is difficult to see. Most of the chloroform used in hospitals is made in Great Britain from methylated spirit.

